# Comparison of Diffusion Kurtosis Imaging and Amide Proton Transfer Imaging in the Diagnosis and Risk Assessment of Prostate Cancer

**DOI:** 10.3389/fonc.2021.640906

**Published:** 2021-04-15

**Authors:** Huijia Yin, Dongdong Wang, Ruifang Yan, Xingxing Jin, Ying Hu, Zhansheng Zhai, Jinhui Duan, Jian Zhang, Kaiyu Wang, Dongming Han

**Affiliations:** ^1^ Department of MR, The First Affiliated Hospital, Xinxiang Medical University, Weihui, China; ^2^ Department of Radiology, People’s Hospital of Zhengzhou, Zhengzhou, China; ^3^ MR Research China, GE Healthcare, Beijing, China

**Keywords:** diffusion kurtosis imaging, amide proton transfer, prostate cancer, Gleason score, benign prostatic hyperplasia

## Abstract

**Objectives:**

This study aims to evaluate and compare the diagnostic value of DKI and APT in prostate cancer (PCa), and their correlation with Gleason Score (GS).

**Materials and Methods:**

DKI and APT imaging of 49 patients with PCa and 51 patients with benign prostatic hyperplasia (BPH) were collected and analyzed, respectively. According to the GS, the patients with PCa were divided into high-risk, intermediate-risk and low-risk groups. The mean kurtosis (MK), mean diffusion (MD) and magnetization transfer ratio asymmetry (MTRasym, 3.5 ppm) values among PCa, BPH, and different GS groups of PCa were compared and analyzed respectively. The diagnostic accuracy of each parameter was evaluated by using the receiver operating characteristic (ROC) curve. The correlation between each parameter and GS was analyzed by using Spearman’s rank correlation.

**Results:**

The MK and MTRasym (3.5 ppm) values were significantly higher in PCa group than in BPH group, while the MD value was significantly lower than in BPH group. The differences of MK/MD/MTRasym (3.5 ppm) between any two of the low-risk, intermediate-risk, and high-risk groups were all statistically significant (p <0.05). The MK value showed the highest diagnostic accuracy in differentiating PCa and BPH, BPH and low-risk, low-risk and intermediate-risk, intermediate-risk and high-risk (AUC = 0.965, 0.882, 0.839, 0.836). The MK/MD/MTRasym (3.ppm) values showed good and moderate correlation with GS (*r* = 0.844, −0.811, 0.640, p <0.05), respectively.

**Conclusion:**

DKI and APT imaging are valuable in the diagnosis of PCa and demonstrate strong correlation with GS, which has great significance in the risk assessment of PCa.

## Introduction

Prostate cancer (PCa) is the second most common cancer in men and the second leading cause of cancer death (1), with its incidence continuing to rise (2). Prostate cancer often occurs simultaneously with benign prostatic hyperplasia and has similar clinical symptoms. Most patients are already in advanced stage of PCa at the time of treatment (2). Therefore, early accurate diagnosis and evaluation of the aggressiveness of PCa is of great significance (3). The Gleason scoring (GS) system is the golden standard for the diagnosis of PCa (4) with a form of the main structural type + secondary structural type according to the degree of differentiation of the glands in the tumor and its growth in the interstitial. GS is an important index to reflect the risk and biological aggressiveness of PCa (4, 5). The higher the GS is, the higher the risk and aggressiveness are (4). Moreover, the treatment strategies and prognosis of PCa vary according to different GSs (6). The GS of PCa is commonly obtained through transrectal ultrasound (TRUS)-guided biopsy, but the biopsy is invasive and easy to reduce the lesions grade or miss small lesions (7). Therefore, for male patients with clinically suspected PCa, it is necessary to find a non-invasive method to accurately diagnose and evaluate its risk and aggressiveness.

In recent years, multi-parametric magnetic resonance imaging (MRI) has gained ascending interest in the management of PCa. Conventional T2WI mainly reflects the contrast information of tissue structure and T2 relaxation characteristics. With high resolution, T2WI is often used in the anatomical division of prostate and the detection of PCa. Conventional diffusion weighted imaging (DWI) is based on Gaussian distribution model, reflecting the diffusion restriction of water molecules by detecting the Brownian motion of water molecules within different tissues *in vivo* (8). Many previous studies concerning DWI (9–11) have been carried out on PCa and shown their potential values in differentiating PCa and noncancerous tissue. However, the movement of water molecules in tissue is often affected by the density of cells, the integrity of cell membrane and the surrounding microenvironment. Diffusion kurtosis imaging (DKI), firstly proposed by Jensen et al. (12) in 2005, is a non-Gaussian diffusion model that reflects microstructural complexity of tumor tissue and is prior to the single index model (13, 14). The two commonly-used parameters derived from DKI are mean kurtosis (MK) and mean diffusivity (MD). The MK value can well represent the deviation degree from Gaussian distribution and reflect the complexity of organizational structure (15). The MD value provides novel diffusion properties that describe the tissue microstructure. Previous studies have shown that DKI can evaluate the aggressiveness of peripheral zone cancer, and its diagnostic value is superior to conventional DWI (16). In addition, some studies indicated that MK value can effectively differentiate the GS of PCa (17, 18). Therefore, DKI can detect the changes of microenvironment in tissue through non-Gaussian distribution model and reflect the invasiveness of PCa. Amide proton transfer weighted imaging (APTWI) is a new technique based on chemical exchange saturation transfer imaging, as well as a novel endogenous contrast mechanism for MRI by detecting low-concentration solutes such as mobile proteins and peptides in tissues or tumors that contain abundant amide (–NH) chemical constituents (19, 20). Zhou et al. (19) detected the macromolecules and peptides *in vivo* by APTWI for the first time. Previous studies have shown that APT is valuable in evaluating the tumor invasiveness (21–23). In addition, a preliminary study of APT on PCa showed that APT can distinguish prostate cancer tissue from non-cancerous peripheral zone tissue (24). Takayama et al. (25) applied APT in GS assessment of prostate cancer, and concluded that there was no correlation between MTRasym (3.5 ppm) and GS. Recent studies (26, 27) also indicated that APT has the potential of detecting active malignant glioma as a non-invasive examination.

However, there are few systematic studies of APT and DKI on the diagnostic assessment and invasive evaluation of PCa without exogenous contrast agents. The purpose of this study is to explore the value of DKI and APT in the diagnosis of PCa, as well as in the evaluation of the aggressiveness of PCa, in order to improve and guide the diagnosis and treatment of PCa in clinical practice.

## Materials and Methods

### Patients

**Figure 1:**
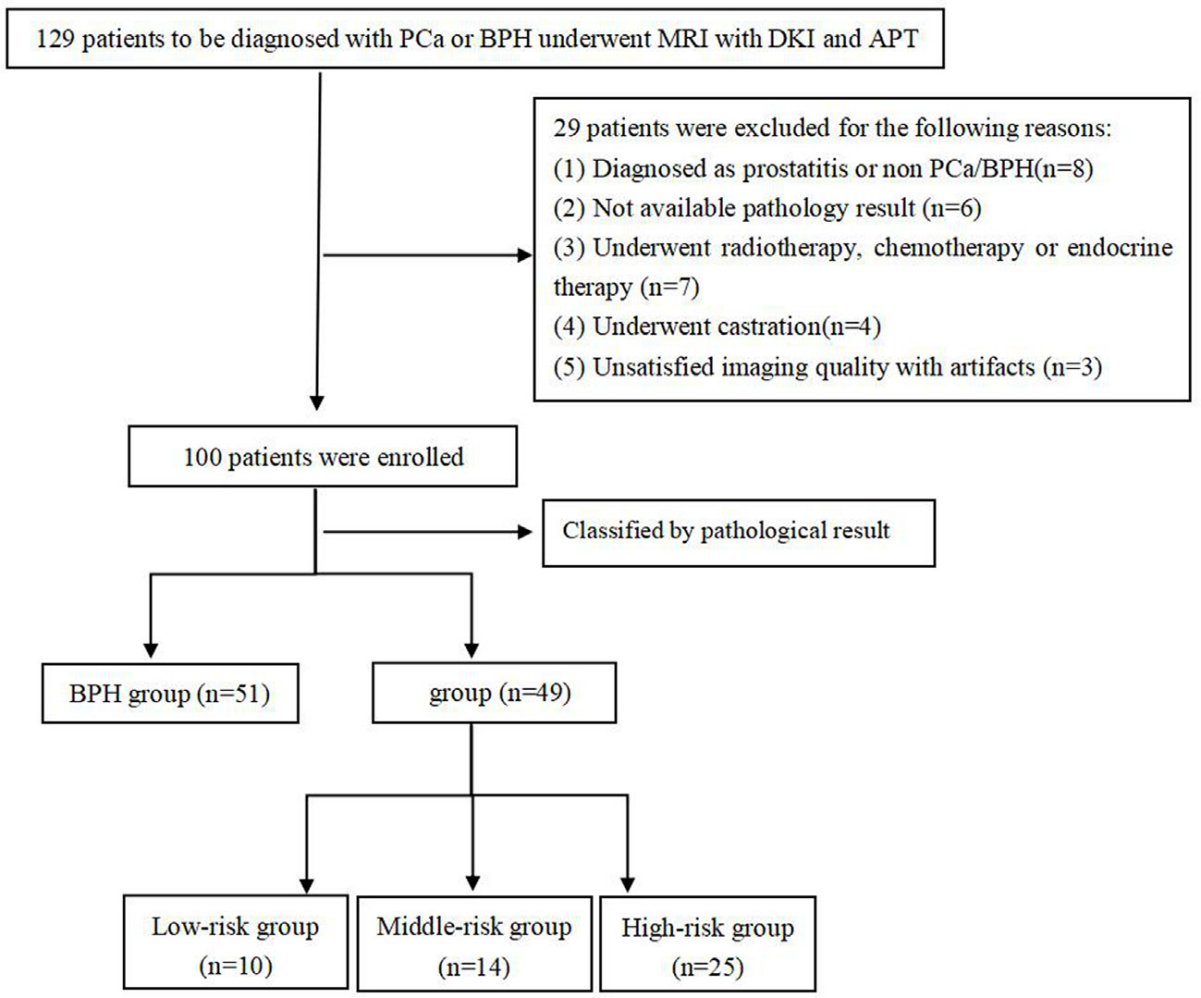
Flowchart of the patient selection process.

This study was approved by the local Institutional Ethics Committee, and all subjects signed the informed consent. From May 2018 to July 2019, 129 patients suspected of PCa and BPH were initially enrolled. Patients were included based on the following criteria: (1) with urinary symptoms and clinical suspicion of prostate cancer or benign prostatic hyperplasia that had not been previously treated; (2) with high level of prostate specific antigen than normal; (3) had no contraindication to MR examinations. Among them, 29 patients were excluded for the following reasons: (1) were non-PCa and non-BPH confirmed by pathological examination (n = 8); (2) did not undergo pathological biopsy or operation (n = 6); (3) received radiotherapy and chemotherapy or endocrine treatment before examination (n = 7); (4) received androgen deprivation therapy before examination (n = 4); (5) could not meet the requirements of post-processing (n = 3) (**Figure 1**). Finally, 49 patients with PCa and 51 patients with BPH were eligible for this study, and their images and pathological data were collected. All GSs were derived from the pathological results after biopsy or radical prostatectomy.

### MRI Protocol

All patients underwent conventional MRI and APT/DKI examinations of prostate on 3.0 T MR scanner (Discovery MR750, GE Healthcare, Milwaukee, Wisconsin) with a 32-channel phased-array torso coil before operation or biopsy. Before the examination, the patients were required to empty the intestines and keep the bladder moderately full. The scanning position is supine position with feet entering the scanner first, and the scan range is from anterior superior iliac spine to upper margin of the pubic symphysis. First, conventional prostate MR images were obtained. Conventional MRI includes the following sequence: coronal/sagittal T2-weighted imaging (T2WI), axial fat-suppressed T2WI, axial T1-weighted imaging (T1WI) without fat suppression, and axial DWI (b = 0, 1,000 mm^2^/s). Under the guidance of the conventional sequence, the DKI (b = 400, 800, 1,200, 1,600 and 2,000 s/mm^2^) and APTWI were performed with the same thickness and spacing. Two-dimensional axial APT imaging was performed using a single-shot echo-planar imaging sequence. According to the tumor area displayed on T2WI, we performed multiple single-slice APT scans, and obtained the corresponding APT information of each slice to draw ROIs for measurements. In addition, any form of contrast-enhanced examination should not be given to patients 24 h before the APTWI scanning to avoid interference with the APT signal (28). **Table 1** displayed the scan parameters.

**Table 1:** Scanning parameters.

Parameters	Ax-T1WI	Ax-T2WI (FS)	DWI	DKI	APT
Sequence	FSE	FSE	SS-EPI	SS-EPI	EPI
TR/TE (ms)	605/8	5,455/109	6,000/60.5	2,500/75.7	3,000/13.6
ETL	5	14	/	/	/
FOV (cm^2^)	38	36 × 36	36 × 36	28 × 28	28 × 28
No. of slices	20	20	20	20	1
Slice thickness(mm)	5.0	5.0	5.0	5.0	5.0
Matrix	320 × 224	320 × 224	128 × 128	128 × 128	128 × 128
Bandwidth (Hz/pixel)	62.50	83.33	250	250	250
NEX	1	1	1, 4	2	1
b-values (s/mm^2^)	–	–	0, 1,000	400, 800, 1,200,1,600, 2,000	–
Saturation pulse/time(only APTWI)	–	–	–	–	2.0 μT, 500 ms
Scan time	1 min 57 s	1 min 33 s	1 min 24 s	5 min 28 s	2 min 36 s
Frequency list (only APTWI)	5,000, 5,000, 5,000, ± 600, ± 575, ± 550, ± 525, ± 500, ± 475, ± 450, ± 425, ± 400, ± 375, ± 350, ± 325, ± 300, ± 275, ± 250, ± 225, ± 200, ± 175, ± 150, ± 125, ± 100, ± 75, ± 50, ± 25 Hz (52 frequencies in total)				

FS, fat suppressed; FSE, fast spin echo; SS-EPI, single-shot echo planar imaging; TR/TE, repetition time/echo time; FOV, field of view; NEX, number of excitations; ETL, echo train length.

In the APT model, the only parameter of APT is the asymmetric magnetization transfer rate at 3.5 ppm (MTRasym (3.5 ppm)). The data were acquired with 52 frequencies, including 49 frequency offsets from 600 to −600 Hz with an interval of 25 Hz and three unsaturated images at 5,000 Hz for signal normalization. With a weak and short B1 power, a Z-spectrum dominated by direct saturation was generated and provided sub-Hertz accuracy for spectral frequency alignment. The formula is MTRasym (3.5 ppm) = (S(−3.5 ppm) − S(+ 3.5 ppm))/S0, where S0 is the signal strength before the application of saturation pulse, S is the signal strength after the application of saturation pulse at a certain chemical shift.

In the DKI model, there are a few parameters. We selected two representative parameters (MK and MD values) to analyze. MK value is the apparent kurtosis coefficient (dimensionless), and MD value is the corrected apparent diffusion parameter (10^−3^ mm^2^/s).

### Image Analysis

All images were transferred to the workstation (Advantage workstation 4.6, GE Healthcare, Milwaukee, Wisconsin) and processed with corresponding software package (DKI, APT). Two experienced urogenital radiologists (H.J.Y. and R.F.Y. with 5 and 15 years of experience, respectively) measured the MTRasym (3.5 ppm), MD, and MK values in a double-blinded manner without knowing the clinical data. In-depth discussions were required if any disagreement occurred, and final agreements were reached by the corresponding author (D.M.H., with more than 20 years of experience in prostate MR imaging). The lesions in the PCa group were confirmed by using T2WI and DWI, and central glands with diffuse hyperplasia were selected for measurements in the BPH group. The DKI/APT images were fused with the T2WI image, the ROIs were drawn slice by slice according to the tumor boundary displayed by T2WI, and the average value of each tumor in different slices was taken as the final results. The principle of the delineation of ROIs were as follows: set the appropriate ROIs at the range of 50–150 mm^2^ according to the size of the lesion; contain solid components as much as possible, but keep a certain distance from the edge of the lesion to avoid volume effect; avoid cystic change, necrosis, calcification and urethra when place the ROIs.

### Statistical Analysis

All statistical analyses were analyzed with SPSS 24.0 (SPSS, Chicago, IL, USA) and MedCalc version 15.6.1 for Windows (MedCalc software, Mariakerke, Belgium). The intraclass correlation coefficient (ICC) was used to assess the consistency of each parameter measured by the two radiologists, and standards are as follows: 0.80–1.00, excellent agreement; 0.60–0.79, good agreement; 0.40–0.59, moderate agreement; 0.20–0.39, fair agreement; and 0.00–0.19, poor agreement (29). Kolmogorov–Smirnov test was used to evaluate whether the distribution of the measured data followed the normal distribution, and the data were expressed as mean ± standard deviation (SD). The difference of MK, MD and MTRasym (3.5 ppm) between PCa group and BPH group was measured by student *t-*test. The difference of MK, MD and MTRasym (3.5 ppm) among BPH and PCa in low, intermediate and high risk groups were tested by analysis of variance (ANOVA), and then the following comparisons between groups were performed by using Student–Newman–Keuls. The receiver operating characteristic (ROC) curve analysis was used to evaluate the diagnostic performance of each parameter. The threshold, sensitivity and specificity were calculated by using the maximum Youden’s index, and the area under the curve (AUC) was compared by using Delong method (30). The correlation between GS and each parameter was analyzed by using Spearman’s correlation analysis, and standards are as follows: r ≥0.75, good; 0.50 ≤ r < 0.75, moderate; 0.25 ≤ r < 0.50, mild; and r <0.25, little or none (31). *P <*0.05 indicated statistical significance.

## Results

**Figure 2:**
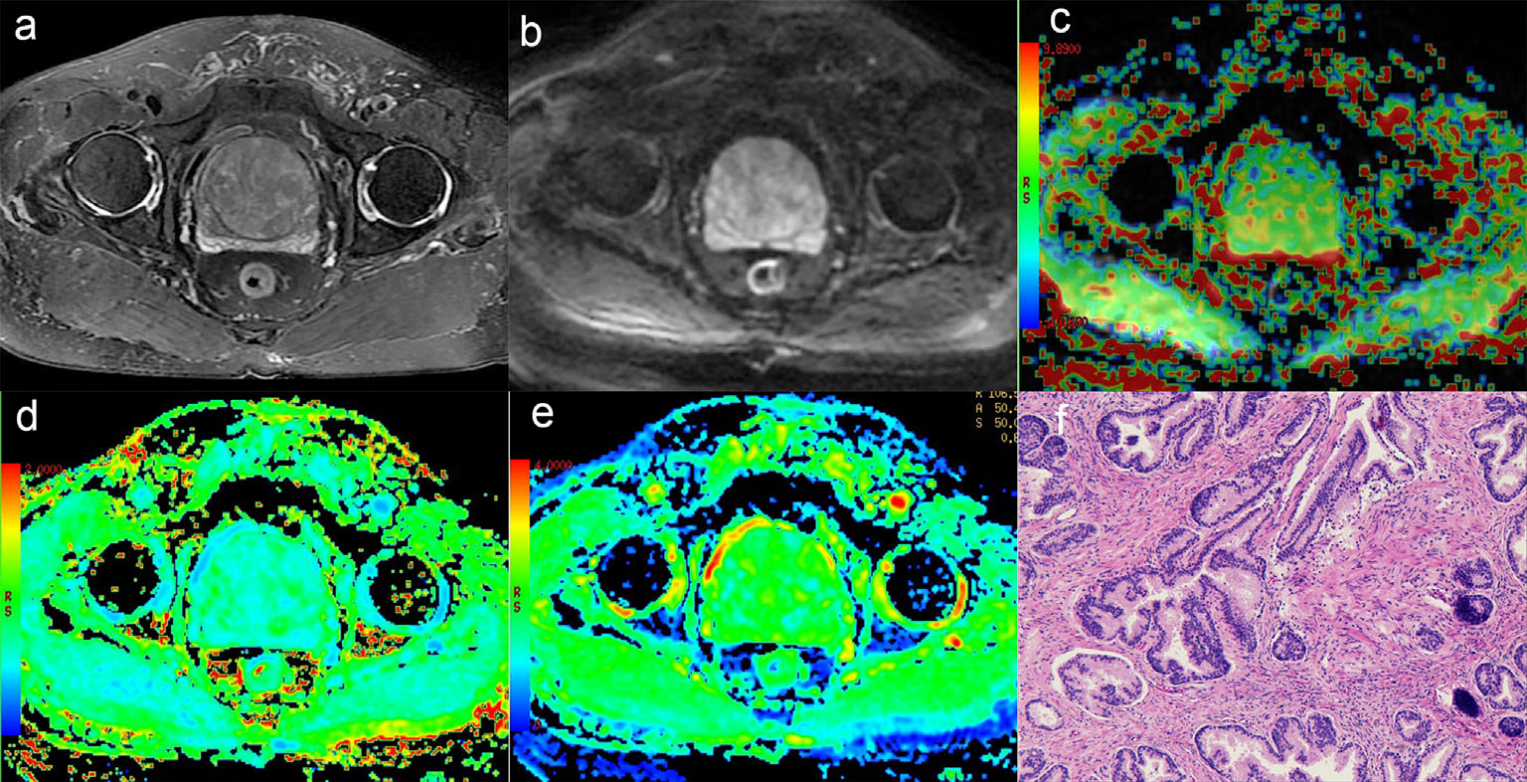
A 75-year-old man with BPH. **(A)** T2-weighted image, **(B)** Diffusion-weighted image, **(C)** APT pseudo-colored map, **(D)** Mean kurtosis pseudo-colored map, **(E)** Mean diffusivity pseudo-colored map, **(F)** pathological image.

**Figure 3:**
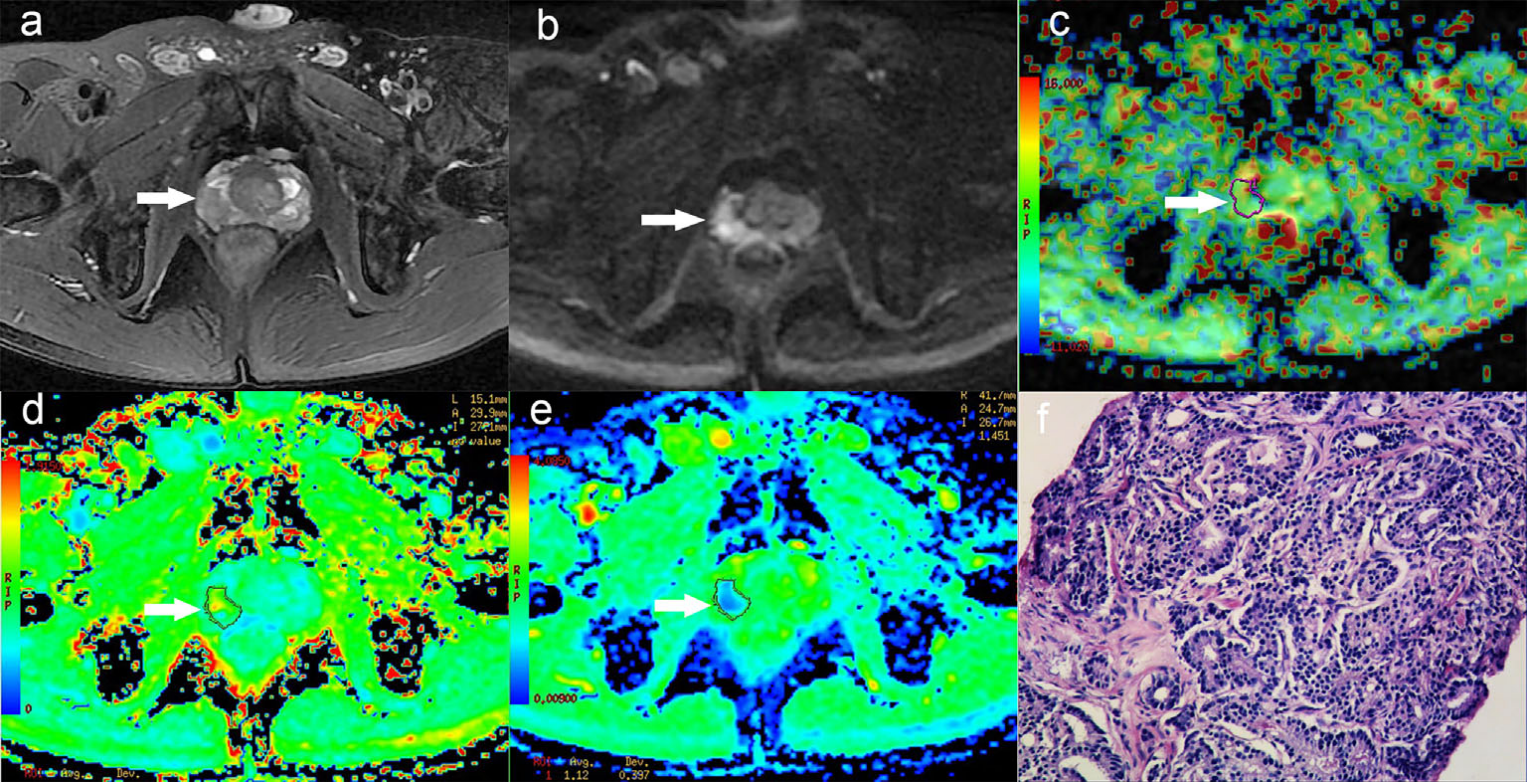
A 58-year-old man with PCa in right peripheral zone, GS is 8. **(A)** T2-weighted image shows hypointense signal in the lesion, **(B)** Diffusion-weighted imaging indicates hyperintense signal, **(C)** APT pseudo-colored map shows yellow-green pseudocolor in the lesion, **(D)** Mean kurtosis pseudo-colored map indicates red-yellow-green pseudocolor in the lesion, **(E)** Mean diffusivity pseudo-colored map shows blue-green pseudocolor in the lesion, **(F)** pathological image.

In this study, 49 patients with PCa and 51 Patients with BPH were enrolled. According to GS, PCa group was divided into low risk group (GS <7), intermediate risk group (GS = 7) and high risk group (GS >7), which included 10 cases in the low-risk group, 14 cases in the intermediate-risk group, 25 cases in the high-risk group (12 cases for GS = 8; seven cases for GS = 9; six cases for GS = 10). There was no significant difference in age among groups, as shown in **Table 2**. The images of derived parameters of DKI and APTWI are shown in **Figures 2** and **3**.

**Table 2:** Characteristics of patients and lesions.

Characteristics	BPH (n = 51)	PCa (n = 49)	*P*-value	Low-risk (n = 10)	Intermediate-risk (n = 14)	High-risk (n = 25)	*P*-value
Patient characteristic							
Age(years)	71.16 ± 9.51	72.86 ± 7.23	0.319	72.20 ± 6.46	74.79 ± 6.80	72.04 ± 7.90	0.512
Serum PSA(ng/ml)							
<4	12	0		0 (10)	0	0	
4–10	17	5		2 (10)	1	2	
>10	22	44		8 (10)	13	23	
Biopsy/ prostatectomy	28/23	29/20	0.179	6/4	8/6	12/13	0.808
Parameters							
MK	0.83 ± 0.10	1.07 ± 0.09	0.000	0.97 ± 0.05	1.04 ± 0.05	1.13 ± 0.07	0.000
MD	1.37 ± 0.43	0.79 ± 0.65	0.000	0.86 ± 0.03	0.81 ± 0.04	0.75 ± 0.06	0.000
MTRasym	2.84 ± 0.49	3.48 ± 0.20	0.000	3.24 ± 0.24	3.46 ± 0.11	3.59 ± 0.11	0.000

### Observer Consistency

The results of ICC showed that the MK, MD and MTRasym (3.5 ppm) values both in PCa and BPH measured by the two observers had good agreement, which is shown in **Table 3**. The ICCs of PCa were 0.882 for MK, 0.873 for MD, and 0.793 for MTRasym (3.5ppm). The ICCs of BPH were 0.895 for MK, 0.879 for MD, and 0.807 for MTRasym (3.5ppm). The average value of each parameter obtained by the two observers was used as the final evaluation.

**Table 3:** Interobserver agreement for each parameter of PCa and BPH.

Parameters	BPH : MK	BPH : MD	BPH : MTRasym (3.5 ppm)	PCa : MK	PCa : MD	PCa : MTRasym (3.5 ppm)
ICC	0.895	0.879	0.807	0.882	0.873	0.793
95%CI	0.824–0.939	0.797–0.929	0.685–0.885	0.800–0.932	0.786–0.927	0.660–0.878

### Comparative Analysis of Parameters

**Figure 4:**
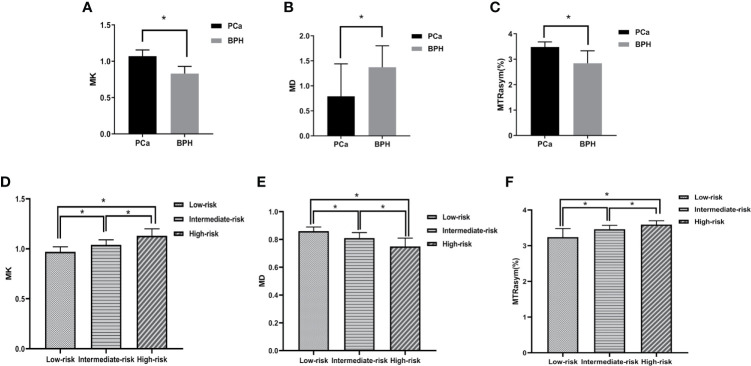
Boxplots of MK/MD/MTRasym (3.5 ppm) values in different groups. **(A–C)** show the comparison of parameters between BPH and PCa, respectively. **(D–F)** show the comparison of parameters between low-, intermediate-, high-risk groups, respectively. * represents P < 0.05.

The MK and MTRasym (3.5 ppm) values in PCa group were significantly higher than those in BPH group, while the MD values in PCa group were significantly lower than those in BPH group (*P <*0.001). The comparisons among different risk groups of PCa showed that the MK and MTRasym (3.5 ppm) values in the low-, intermediate- and high-risk groups increased gradually (*P <*0.001). And the MD values in the low-, intermediate- and high-risk groups decreased gradually (*P <*0.001). The differences of MK, MD and MTRasym (3.5 ppm) values among all subgroups were statistically significant (*P <*0.001). The results are shown in **Table 2** and **Figure 4**.

### ROC Analysis

**Figure 5:**
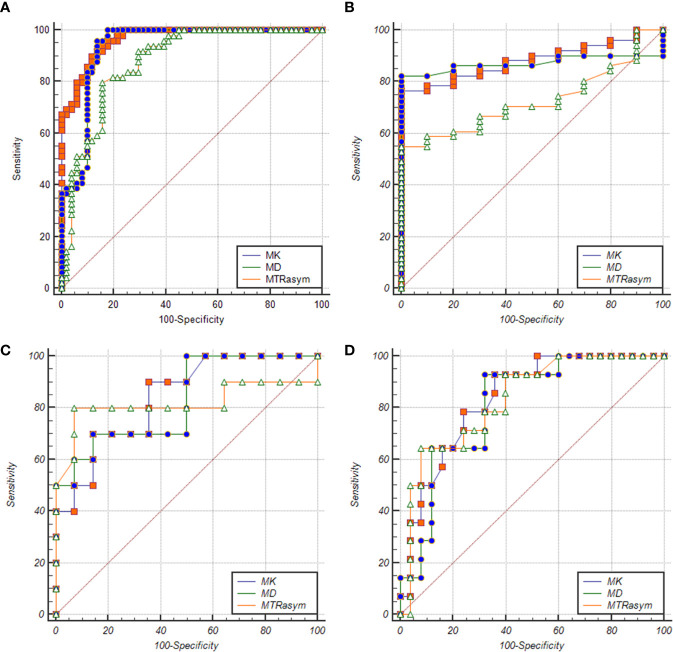
ROC curves of MK/MD/MTRasym (3.5 ppm) values between BPH and PCa groups **(A)**, BPH and low-risk groups **(B)**, low-risk and intermediate-risk groups **(C)**, intermediate-risk and high-risk groups **(D)**, respectively.

The ROC curve of MK, MD, MTRasym (3.5 ppm) values in the diagnosis of PCa and different risk groups is shown in **Figure 5**. For all the groups, the area under curve (AUC) of MK value is the highest (AUC = 0.965, 0.882, 0.839, 0.836). The comparison between the intermediate-risk and high-risk groups showed that AUC (MK) > AUC (MTRasym (3.5 ppm)) > AUC (MD). But in the comparisons between other groups, AUC (MK) > AUC (MD) > AUC (MTRasym (3.5 ppm)). In the ROC analysis, the differences among the AUC (MK), AUC (MD) and AUC (MTRasym (3.5 ppm)) were statistically significant for all groups (*P <*0.001). The details are summarized in **Table 4**.

**Table 4:** Parameters of ROC curve.

Group	Parameters	AUC (95%CI)	p value	Threshold	Sensitivity (%)	Specificity (%)	Youden’s index
**PCa–BPH**	**MK**	0.965 (0.907–0.991)	<0.0001	0.9325	95.92	82.35	0.7827
	**MD**	0.934 (0.866–0.974)	<0.0001	0.9025	100.00	82.35	0.8235
	**MTRasym**	0.877 (0.832–0.968)	<0.0001	3.35	79.59	84.31	0.6391
**BPH-** **Low Risk Group**	**MK**	0.882 (0.774–0.951)	<0.0001	0.902	76.47	100.00	0.7647
	**MD**	0.873 (0.762–0.944)	<0.0001	0.9025	82.35	100.00	0.8235
	**MTRasym**	0.727 (0.598–0.834)	<0.0001	2.93	54.90	100.00	0.5490
**Low-** **Middle Risk Group**	**MK**	0.839 (0.633–0.956)	<0.0001	0.991	70.00	85.71	0.5571
	**MD**	0.829 (0.621–0.950)	<0.0001	0.85	70.00	85.71	0.5571
	**MTRasym**	0.818 (0.608–0.944)	<0.0001	3.35	80.00	92.86	0.7286
**Middle-High Risk Group**	**MK**	0.836 (0.682–0.935)	<0.0001	1.098	92.86	64.00	0.5686
	**MD**	0.811 (0.654–0.919)	<0.0001	0.773	92.86	68.00	0.6086
	**MTRasym**	0.831 (0.677–0.932)	<0.0001	3.455	64.29	92.00	0.5629

PCa, prostate cancer; BPH, benign prostatic hyperplasia; MK, mean kurtosis; MD, mean diffusion; MTRasym, magnetization transfer ratio asymmetry.

### Correlation Analysis

**Figure 6:**
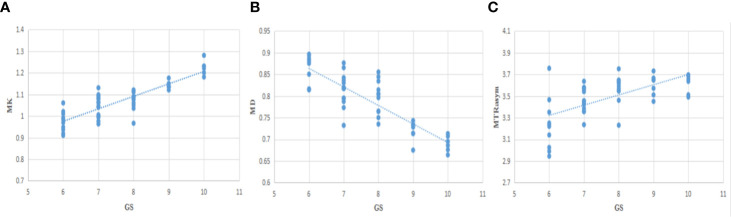
The correlation between MK/MD/MTRasym (3.5 ppm) and Gleason Scores (GS) **(A−C)**, r=0.844, −0.811, 0.640, respectively.

The correlation between each parameter derived from DKI and APT and GS of PCa patients was analyzed. MK value shows a good positive correlation with GS (*r* = 0.844, *P <*0.001), MD shows a good negative correlation with GS (*r* = −0.811, *P <*0.001), and MTRasym (3.5 ppm) was moderately and positively correlated with GS (*r* = 0.640, *P <*0.001). The relationship is shown in **Figure 6**.

## Discussion

In our study, the diagnostic value of DKI and APT for prostate cancer (PCa), as well as the risk assessment of PCa by using DKI and APT were analyzed. According to the results of the study, MTRasym (3.5 ppm), MK and MD can be used to distinguish prostate cancer from BPH. Meanwhile, these three parameters shows ability in the risk assessment of prostate cancer, which is consistent with previous research results (16–18, 24, 25).

This study indicated that MTRasym (3.5 ppm) value in PCa was generally higher than that in BPH. The main conclusion was consistent with the conclusion of Jia et al. (24), which was that APTWI has the potential to discriminate between cancer and noncancerous tissues. The technical reason was that APT can detect the change of mobile protein and polypeptide *in vivo* noninvasively. And the pathology basis was that normal prostate tissue is composed of loose glands, large extracellular spaces and glands cavity filled with fluid (4, 32), while in PCa tissue, the cell arrangement is tight, the intercellular space is reduced, the volume of gland cavity is reduced, and the macromolecules and polypeptides secreted by tumor tissue are increased (33). Our study also found that MK value was higher and MD value was lower in PCa than that in BPH. The main case was that in cancerous tissue the gland structure is destroyed, the cell density increases, and then the complexity of prostate microstructure increases, and the diffusion movement of water molecules is more restricted (34).

GS is an internationally recognized scoring system for prostate cancer reference (4, 35), which classifies the risk according to the degree of differentiation of intratumoral glands and their growth pattern in stroma. It is a pathological reference standard and measures the invasiveness of PCa (4, 5, 36). We also compared the MTRasym (3.5 ppm)/MK/MD value among the low-, intermediate- and high-risk groups, concluding that MK and MTRasym (3.5 ppm) values of the low-, medium- and high-risk groups increased gradually, while the MD value decreased gradually. It is speculated that with the increasing of tumor proliferation, the density of tumor cells, the mobile protein content, and diffusion restriction in the lesions are also increased (25).

ROC analysis showed that in the identification of BPH and PCa group, BPH and low-risk group, low-risk group and intermediate-risk group, intermediate-risk group and high-risk group, MK shows the largest AUC among the three used parameters as the AUC of the first three differentiation is MK > MD > MTRasym (3.5 ppm) and AUC of the fourth differentiation is MK > MTRasym (3.5 ppm) > MD. That is to say, MK, MD, and MTRasym (3.5 ppm) all can be used in the risk assessment of PCa, and MK has the greatest diagnostic efficiency for PCa, which is consistent with the conclusions of Suo, Quentin and Tamura (37–39). It is worth mention that in our comparison of PCa and BPH, the high-risk cases account for a large proportion (25/49) in PCa group, which may overestimate the diagnostic efficiency of each parameter between groups.

Our study shows that MK and MTRasym (3.5 ppm) respectively have a good and moderate positive correlation (*r* = 0.844, 0.640) with GS, and MD has a good negative correlation (*r* = −0.811) with GS. The MK has the strongest correlation, then MD the second, and MTRasym (3.5 ppm) the last. This implies that MK value has the strongest ability to predict GS of PCa, which is consistent with the results of Wang et al. and Tamada et al. (17, 18). When it comes to MTRasym (3.5 ppm), the result is consistent with Togao and Zhou et al. (20, 21) in the study of central nervous system tumors and Wu, Li et al. (14, 40) in the study of prostate cancer, while different from the results of Takayama and Barrett et al. (25, 41), with finding that there was no significant correlation between MTRasym (3.5 ppm) value and GS, which can be explained by the following reasons. On one hand, with the increase of GS, tumor cell density and proliferation rate increased gradually, which is the main reason for the increase of MTRasym (3.5ppm) value (21, 24). At the same time, the tumor necrosis area also increased, which may also contribute to the increase of the MTRasym (3.5 ppm) value (21). On the other hand, the gland structure is destroyed, and liquid, mobile protein and polypeptide which contained in the gland is gradually reduced, which may have little influence but is the factor for the decrease of the MTRasym (3.5ppm) value (33, 42). In previous study, Takayama et al. (25) selected lesions with ROI ≥300 mm^2^, which may contain more areas of microcystic necrosis and reduce the MTRasym value of GS >7 cases. However, the ROI selected in our study is smaller, correspondingly there was less invisible cystic change and necrosis. In addition, there was no case with GS = 10 in previous study (22), while in our study, there were more patients in the high-risk group, especially GS = 10, so the MTRasym value of the high-risk group was higher, and MTRasym value has a positive correlation with GS. Moreover, the cell density and tumor heterogeneity of the lesions increased with high proliferation rate, so MK value increased gradually; the cell density increased and the diffusion restriction of water molecules increased, so MD decreased gradually (17, 18, 43). The study of Shan et al. (44) have also indicated that the parameters of DKI can be used to distinguish high- and low-risk prostate cancer. In summary, MK, MD and MTRasym (3.5 ppm) values can be used to evaluate the potential invasion of PCa and have correlations with GS risk.

### Limitations of This Study

There are some limitations in this study. First, the number of cases in each risk group of PCa is small, especially in the low-risk group (10/49), which is the least. Second, the prostate cancer selected in this study has artificially avoided some rare subtypes like urothelial carcinoma and squamous cell carcinoma, which reduces the representativeness of the research results. In the future, we will continue to collect cases and further expand our sample size for more robust analysis. Third, in this study, GS = 7 is not divided into 3 + 4 or 4 + 3 groups for comparative analysis. We will gradually refine the groups for further research. Fourth, the artificial sketch of ROI in this study is with some subjectivity, which may affect the analysis. Moreover, we can’t avoid the invisible necrosis or cystic change totally, which also lead to contamination for our result. Methods like histograms and iconography may be more objective and can be used to improve the accuracy.

## Conclusion

In conclusion, both DKI and APT can be used to diagnose PCa and assess its risk without additional use of external contrast agent, but DKI shows better diagnostic efficiency. They all have the potential to be used in routine clinical practice as new indicators to evaluate the risk of PCa, and to help early diagnosis and personalized diagnosis and treatment of PCa.

## Data Availability Statement

The original contributions presented in the study are included in the article/supplementary material. Further inquiries can be directed to the corresponding author.

## Ethics Statement

The studies involving human participants were reviewed and approved by the local Ethics Committee, the First Affiliated Hospital of Xinxiang Medical University. The patients/participants provided their written informed consent to participate in this study.

## Author Contributions

HY, DW, and DH conceived and designed the study. HY and DW contributed to the manuscript editing preparation. HY and RY participated in literature research. DW and KW performed the statistical analysis. XJ, YH, ZZ, JD, and JZ participated in the clinical studies and collect cases. HY and DW wrote the first draft of the manuscript. DH participated in the most revision work and takes responsibility for the integrity of the data and the accuracy of the data analysis. All authors contributed to the article and approved the submitted version.

## Funding

This work has received funding by the Henan Medical Science and Technology Research Program [grant number LHGJ20190448].

## Conflict of Interest

KW was employed by GE Healthcare.

The remaining authors declare that the research was conducted in the absence of any commercial or financial relationships that could be construed as a potential conflict of interest.
